# *Cis*-[Cr(C_2_O_4_)(pm)(OH_2_)_2_]^+^ Coordination Ion as a Specific Sensing Ion for H_2_O_2_ Detection in HT22 Cells

**DOI:** 10.3390/molecules19068533

**Published:** 2014-06-23

**Authors:** Dagmara Jacewicz, Kamila Siedlecka-Kroplewska, Joanna Pranczk, Dariusz Wyrzykowski, Michał Woźniak, Lech Chmurzyński

**Affiliations:** 1 Faculty of Chemistry, University of Gdańsk, Wita Stwosza 63, Gdańsk 80-308, Poland; E-Mails: joanna.pranczk@phdstud.ug.edu.pl (J.P.); dariusz.wyrzykowski@ug.edu.pl (D.W.); lech.chmurzynski@ug.edu.pl (L.C.); 2 Department of Histology, Medical University of Gdańsk, Dębinki 1, Gdańsk 80-211, Poland; E-Mail: ksiedlecka@gumed.edu.pl; 3 Department of Medical Chemistry, Medical University of Gdańsk, Dębinki 1, Gdańsk 80-211, Poland; E-Mail: mwozniak@gumed.edu.pl

**Keywords:** glutamate, HT-22 cells, hydrogen peroxide, kinetic mechanism, Cr(III) complexes, pyridoxamine

## Abstract

The purpose of this study was to examine the application of the coordinated *cis*-[Cr(C_2_O_4_)(pm)(OH)_2_]^+^ cation where pm denotes pyridoxamine, as a specific sensing ion for the detection of hydrogen peroxide (H_2_O_2_). The proposed method for H_2_O_2_ detection includes two key steps. The first step is based on the nonenzymatic decarboxylation of pyruvate upon reaction with H_2_O_2_, while the second step is based on the interaction of *cis*-[Cr(C_2_O_4_)(pm)(OH_2_)_2_]^+^ with the CO_2_ released in the previous step. Using this method H_2_O_2_ generated during glutamate-induced oxidative stress was detected in HT22 hippocampal cells. The coordination ion *cis*-[Cr(C_2_O_4_)(pm)(OH_2_)_2_]^+^ and the spectrophotometric stopped-flow technique were applied to determine the CO_2_ concentration in cell lysates, supernatants and cell-free culture medium. Prior to CO_2_ assessment pyruvate was added to all samples studied. Pyruvate reacts with H_2_O_2_ with 1:1 stoichiometry, and consequently the amount of CO_2_ released in this reaction is equivalent to the amount of H_2_O_2_.

## 1. Introduction

Reactive oxygen species (ROS) generation contributes to the ethiology of many diseases, including diabetes, arteriosclerosis or neurodegenerative disorders and others [1,2,3]. The increase in intracellular production of free radicals may lead to cellular damage, including alterations of lipids, proteins and DNA [4]. ROS [5,6], in particular superoxide anion (O_2_^•−^), hydroxyl radical (OH^•^) or hydrogen peroxide (H_2_O_2_) and reactive nitrogen species [7,8,9,10], e.g., nitrogen dioxide, are highly cytotoxic. Superoxide anion is able to react with nitric oxide to form toxic peroxinitrite anions or dismutate into H_2_O_2_ which in turn can be transformed into highly reactive and toxic hydroxyl radicals. H_2_O_2_ is one of the most important mediators of oxidative stress detected under pathological conditions. It was observed that under pathological conditions such as ischemia-reperfusion injury, excessive production of H_2_O_2_ occurs. H_2_O_2_ is probably involved in the neuronal damage seen in Parkinson’s and Huntington’s diseases and other neuronal disorders.

Oxidative stress can be evaluated by detecting ROS using biosensors [11]. However, in most cases, the concentration of ROS is evaluated without enough precision [12,13,14]. The other limitations include not enough sensitivity or lack of specificity. Therefore, there is a need for developing more specific, sensitive and effective methods for the detection and measurement of intracellular ROS levels [12]. Pyruvic acid, like other α-ketoacids, acts as a H_2_O_2_ scavenger and is able to react non-enzymatically with H_2_O_2_ yielding the following products: acetic acid, H_2_O and CO_2_. Interestingly, pyruvate is present in mammalian cells and its antioxidative properties contribute to the cellular defense against H_2_O_2_-mediated cytotoxicity [15]. In the present study, a new method of the H_2_O_2_ concentration assessment has been demonstrated. This method is based on the ability of the molecular biosensor—coordinate ion *cis*-[Cr(C_2_O_4_)(pm)(OH)_2_]^+^ to effectively trap CO_2_, one of the final products of the chemical reaction between H_2_O_2_ and exogenous pyruvate (1). Noteworthily, the amount of CO_2_ released in this reaction is equivalent to the amount of H_2_O_2_:

pyruvate + H_2_O_2_ → acetate + CO_2_ + H_2_O (1)

Glutamate is a neurotransmitter in the central nervous system. It was reported to induce neuronal cell death at mM levels of concentrations [16]. High concentrations of extracellular glutamate inhibit the glutamate/cystine antiporter, which results in the depletion of intracellular glutathione that converts H_2_O_2_ to H_2_O [17]. Typically, 5 mM glutamate was shown to induce oxidative stress in HT22 cells leading to death [18]. Therefore, as an experimental model to study the generation of H_2_O_2_, mouse hippocampal HT22 cells treated with 5 mM l-glutamate were used in our study.

Previously, our research group reported a quantitative determination of H_2_O_2_ in osteosarcoma cells [19]. In this method a molecular biosensor was used, the most effective being a coordination complex ion *cis*-[Cr(C_2_O_4_)(pm)(OH_2_)_2_]^+^. The CO_2_ uptake was studied using a spectrophotometric stopped-flow method whereby it was possible to determine the content of H_2_O_2_ in the biological material under anaerobic conditions. The method described is based on the assumption of the selective reaction of α-keto acid—pyruvate with H_2_O_2_, with the subsequent decarboxylation of the intermediate product, pyruvic peracid, and the capture of the CO_2_ released. The results provided arguments for the usefulness of pyruvate application for cell culture studies where culture media could produce significant levels of H_2_O_2_ before treatment of cells. On the other hand endogenous and exogenous sources of H_2_O_2_ implicated in cytotoxity in a variety of human diseases can be safely prevented by pyruvate. The efficiency of this scavenger was clearly demonstrated by a novel application of a molecular CO_2_ detection method based on *cis*-[Cr(C_2_O_4_)(pm)(OH_2_)_2_]^+^ ion.

## 2. Results and Discussion

The *cis*-[Cr(C_2_O_4_)(pm)(OH_2_)_2_]^+^ ion was previously developed as a specific molecular biosensor to detect uptake of CO_2_, generated in the reaction between Na_2_CO_3_ and HCl [20]. To do this the reaction between the *cis*-[Cr(C_2_O_4_)(pm)(OH_2_)_2_]^+^ ion and carbon dioxide in aqueous solution was monitored between 340 nm and 700 nm using a spectrophotometric stopped-flow method. It was observed that the reaction of carbon dioxide uptake by the applied biosensor ran in two-steps. The first step was about 50 times faster than the second one. This conclusion is based on the analytical results. The analytical model was already described in [21]. During the carbon dioxide uptake (where CO_2_ was generated in a chemical reaction) by *cis*-[Cr(C_2_O_4_)(pm)(OH_2_)_2_]^+^ complex ion the most significant changes of absorbance were seen at λ = 560 nm. Consequently, on the basis of results obtained a two-step mechanism for the uptake of carbon dioxide by *cis*-[Cr(C_2_O_4_)(pm)(OH_2_)_2_]^+^ ion was proposed and described [21].

**Figure 1:**
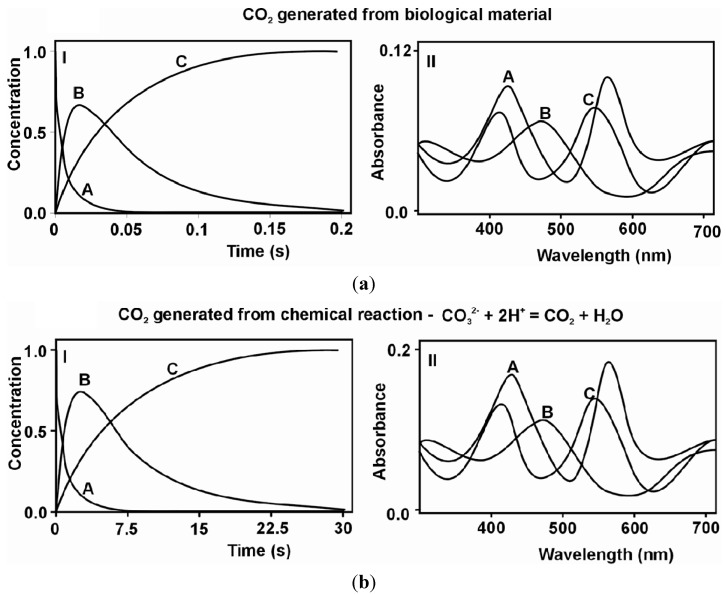
Comparison of the biological and chemical reaction models. (**a**) The biological reaction model (I) Curves of concentration decay and buildup of the substrate A (which is the *cis*-[Cr(C_2_O_4_)(pm)(OH_2_)_2_]^+^ ion), product C as *cis-*[Cr(C_2_O_4_)(pm)(O_2_CO)]^−^ ion, and intermediate product B. (II) Absorption spectra of the reactants A, B and C - the detection of CO_2_ generated during glutamate induced oxidative stress in HT22 cells. (**b**) The chemical reaction model (I) Curves of concentration decay and buildup of the substrate A (which is the *cis*-[Cr(C_2_O_4_)(pm)(OH_2_)_2_]^+^ ion), product C as *cis*-[Cr(C_2_O_4_)(pm)(O_2_CO)]^−^ ion, and intermediate product B. (II) Absorption spectra of the reactants A, B and C at pH = 7.13, t = 20 °C.

Having the above knowledge concerning a chemical model, the same coordination compound of Cr(III) with a bidendate ligand—pyridoxamine—was checked and successfully applied in this study also in biological material, namely for the detection of CO_2_ generated during the glutamate-induced oxidative stress in HT22 cells. The results obtained are presented in Figure 1a For comparison, the previously obtained results of global analysis (GA) for reaction of CO_2_ uptake by the *cis*-[Cr(C_2_O_4_)(pm)(OH_2_)_2_]^+^ ion in a chemical model within the consecutive reaction model (A→B→C) are presented in Figure 1b. In Figure 1a,b symbol “A” means the substrate *cis*-[Cr(C_2_O_4_)(pm)(OH_2_)_2_]^+^, “B”- intermediate product, and “C”- the final product *cis-*[Cr(C_2_O_4_)(pm)(O_2_CO)]^−^. The different behaviour of A, B and C is the result of decrease in the substrate concentration and product formation. As seen the same absorption maxima can be observed for both systems – chemical and biological. This conformity can be treated as a confirmation that the proposed chemical model fits the biological system. Figure 1a shows the global analysis results for the reaction of CO_2_ uptake from biological material by the *cis*-[Cr(C_2_O_4_)(pm)(OH_2_)_2_]^+^ ion. Cr(III) is inert and this causes the reaction to be slowed down. To confirm the mechanism of uptake previously proposed on the basis of the chemical reaction system [21] in the biological model, in the first step (carbon dioxide uptake), kinetic data were fitted by a simple A→B reaction model (where B denotes the intermediate). Furthermore, in the second step (the closure of the ring of carbonate ion) [22], the reaction was monitored at the wavelength where the maximum difference in molar absorptivities between the intermediate products and products (B→C reaction model) was observed, at λ = 560 nm (Figure 1). It should be pointed that the results obtained by the global analysis (GA) [23] method were confirmed by another independent method of the singular value decomposition (SVD) [22] analysis (Figure 1). GA and SVD are the mathematical methods used in calculations.

In this study a new method for H_2_O_2_ detection in cell lysates, supernatants and cell-free culture medium is demonstrated. This method is based on both the interaction of the coordination compound *cis*-[Cr(C_2_O_4_)(pm)(OH)_2_]^+^ with CO_2_ and the nonenzymatic reaction of pyruvate with H_2_O_2_. Using *cis*-[Cr(C_2_O_4_)(pm)(OH)_2_]^+^ and the spectrophotometric stopped-flow technique the CO_2_ concentration in cell lysates, supernatants and culture medium was determined. Since pyruvate reacts nonenzymatically with H_2_O_2_ with 1:1 stoichiometry releasing CO_2_, the amount of H_2_O_2_ in this reaction is equivalent to the amount of CO_2_ released. Therefore, prior to CO_2_ assessment pyruvate was added to all samples studied.

**Figure 2:**
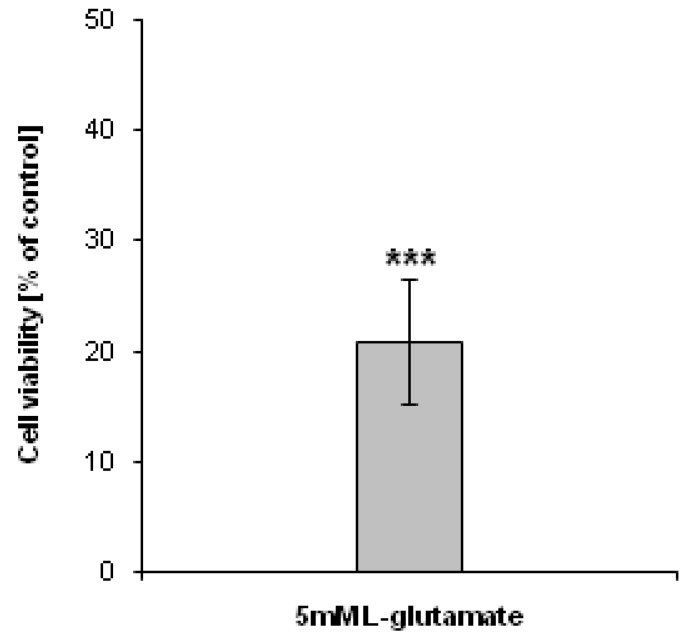
Antiproliferative effect of l-glutamate. HT22 cells were treated with 5 mM l-glutamate for 24 h. The cell viability was assessed using the 3-[4,5-dimethylthiazol-2-yl]-2,5-diphenyltetrazolium bromide (MTT) assay. Data are presented as mean ± SD. *** *p* <0.001, statistically significant differences compared to control (untreated) cells.

As an experimental model to study the generation of H_2_O_2_, mouse hippocampal HT22 cells treated with sodium 5 mM l-glutamate were used. Five mM glutamate was used to induce the oxidative stress in HT22 cells, which will subsequently lead to cell death [18]. In agreement with this data, our results showed that the viability of HT22 cells treated with 5 mM l-glutamate for 24 h decreased dramatically (Figure 2).

**Figure 3:**
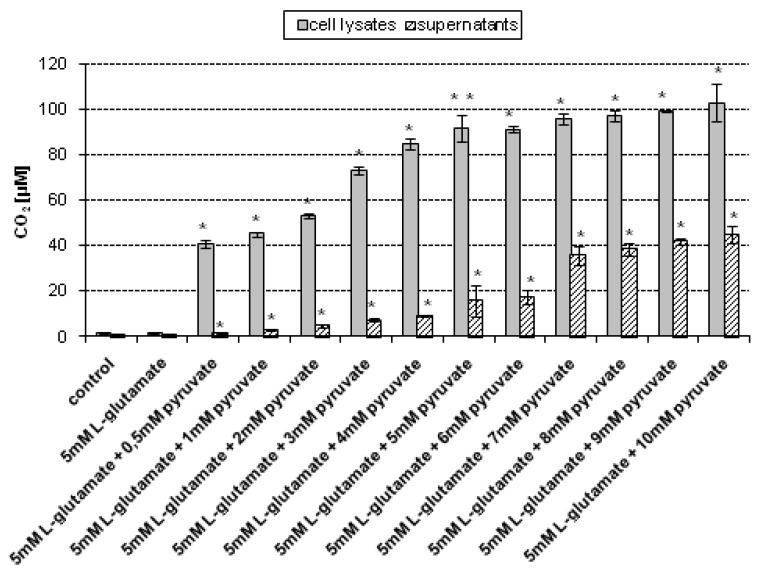
CO_2_ assessment in cell lysates and supernatants upon treatment with 5 mM sodium l-glutamate. HT22 cells were incubated with 5 mM sodium l-glutamate for 24 h. After treatment, cells and superntants were separated. The cells were then lysed using a lysis buffer. Prior to CO_2_ measurement sodium pyruvate was added (final concentations of sodium pyruvate: 0.5–10 mM, respectively) to the samples containing cell lysates and supernatants, respectively. The CO_2_ level was assessed using a stopped-flow technique. Data are expressed as mean ± SD of three independent experiments. * *p* < 0.05, ** *p* < 0.01, statistically significant differences compared to the sample treated with 5 mM l-glutamate alone, without addition of sodium pyruvate; control - untreated cells.

In the experiments, the main source of CO_2_ measured in cell lysates was the reaction of pyruvate with H_2_O_2_ produced endogenously in HT22 cells treated with 5 mM l-glutamate for 24 h. In order to determine the most effective concentration of sodium pyruvate required to scavenge H_2_O_2_ present in cell lysates, the following concentrations of sodium pyruvate were tested: 0.5, 1, 2, 3, 4, 5, 6, 7, 8, 9 and 10 mM. The CO_2_ levels in lysates of control (untreated) cells and lysates of cells treated with 5 mM l-glutamate, measured without the addition of pyruvate, were 1.09 μM (± 0.06) and 1.1 μM (±0.05), respectively. It has been found that upon addition of 0.5–4 mM sodium pyruvate to the lysates of l-glutamate-treated HT22 cells, the CO_2_ level gradually increased (Figure 3).

Noteworthily, at pyruvate concentrations ranging from 5 mM to 10 mM only slight changes in the CO_2_ level measured in the cell lysates were observed—the CO_2_ level did not increase significantly. These results suggest that the 5 mM–10 mM concentration range of sodium pyruvate can be used in the proposed method to effectively assess the CO_2_ concentration in the cell lysates obtained upon lysis of l-glutamate-treated HT22 cells.

H_2_O_2_ has the ability to penetrate biological membranes, which enables it to be released from the cells where it is produced and thus affect neighbouring cells [5]. Therefore, the CO_2_level in supernatants—the surrounding of HT22 cells was assessed. Noteworthily, the source of CO_2_ detected in supernatants can also be H_2_O_2_ production resulting from oxidation of components of culture medium [24]. The results revealed that the CO_2_ levels in supernatants separated from control (untreated) cells and those collected after separation of cells treated with 5 mM l-glutamate, both measured without the addition of pyruvate, were 0.94 μM (± 0.05) and 1.03 μM (± 0.22), respectively. Moreover, it has been found that upon addition of 0.5–6 mM sodium pyruvate to the supernatants separated from l-glutamate-treated HT22 cells, the CO_2_ level gradually increased (Figure 3). At pyruvate concentrations ranging from 7 mM to 10 mM only slight changes in the CO_2_ level in the supernatants were observed—the CO_2_ level did not increased significantly. These results indicate that among the pyruvate concentrations tested, the concentration range 7-10 mM is sufficient to assess the CO_2_ level in the supernatants collected upon separation of HT22 cells treated with 5 mM l-glutamate.

**Figure 4:**
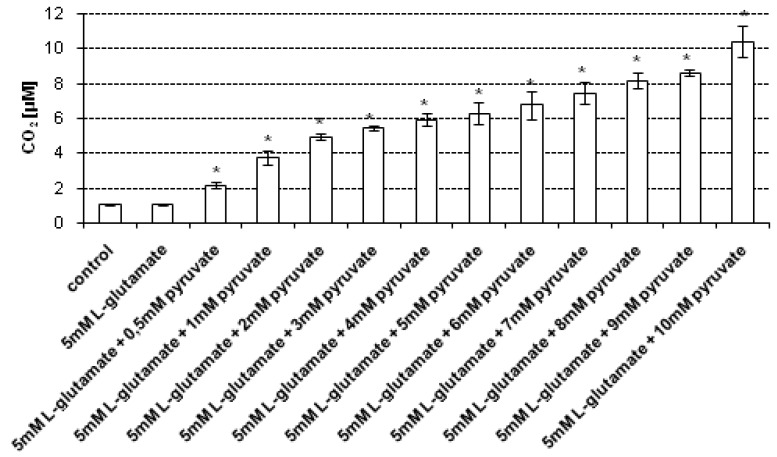
CO_2_ assessment in cell-free complete culture medium upon treatment with 5 mM sodium l-glutamate. The cell-free complete medium was incubated with 5 mM sodium l-glutamate for 24 h. After treatment, sodium pyruvate was added (final concentations of sodium pyruvate: 0.5–10 mM, respectively). The CO_2_ level was assessed using a stopped-flow technique. Data are expressed as mean ± SD. * *p* < 0.05, statistically significant differences compared to the sample treated with 5 mM l-glutamate alone, without addition of sodium pyruvate; control – untreated sample.

As mentioned previously, the culture medium may be a source of H_2_O_2_ generation [24]. Moreover, it is important to evaluate whether the CO_2_ level assessed by our method can be influenced by the HCO_3_^−^/CO_2_ buffer system used in growth media to maintain the proper pH. Therefore, in addition to the CO_2_ assessment in cell lysates and supernatants, the CO_2_ level in cell-free complete culture medium was examined, under conditions of the experiment not resulting from endogenous (cellular) production of H_2_O_2_. The CO_2_ concentration was measured without addition or upon addition of different concentrations of sodium pyruvate, respectively. The culture medium were treated with 5 mM l-glutamate for 24 h and then collected. The CO_2_ concentration in control (untreated) medium sample and those treated with 5 mM l-glutamate, both measured without the addition of pyruvate, were 1.06 μM (± 0.04) and 1.09 μM (± 0.02), respectively. It has been found that upon addition of 0.5–10 mM sodium pyruvate, the CO_2_ level gradually increased (Figure 4). As mentioned previously, one possible explanation is that the culture medium may also be a source of H_2_O_2_ generation [19].

## 3. Experimental Section

### 3.1. Chemicals

l-Glutamic acid monosodium salt monohydrate was purchased from Sigma (St. Louis, MO, USA). l-Glutamic acid monosodium salt solutions were prepared prior to use in sterile physiological saline solution. Sterile sodium pyruvate solution (100 mM) was obtained from Sigma and diluted in sterile water to desired concentrations before use. Dihydrochloride pyridoxamine was purchased from Sigma.

### 3.2. Reagents

The *cis*-[Cr(C_2_O_4_)(pm)(OH_2_)_2_]^+^ ion was prepared according to standard literature procedures [19]. The final products, *cis*-[Cr(C_2_O_4_)(L-L)(O_2_CO)]^−^ (where L-L denotes bidentate ligand—pyridoxamine (pm)) was synthesised by the previously reported method [21]. The complex *cis*-[Cr(C_2_O_4_)(pm)(OH_2_)_2_]^+^ was synthesised from K[Cr(C_2_O_4_)(H_2_O)_2_]·3H_2_O and pyridoxamine.

### 3.3. Cell Culture

The hippocampal neuronal HT22 cell line was kindly provided by Professor T. Grune (Institute of Biological Chemistry and Nutrition, University Hohenheim, Stuttgart, Germany). HT22 cells were maintained at 37 °C in a humidified atmosphere containing 5% (v/v) CO_2_ in Dulbecco’s Modified Eagle’s Medium without sodium pyruvate (Sigma), supplemented with 10% (v/v) heat-inactivated fetal bovine serum (Sigma), 100 IU/mL penicillin (Sigma) and 100 μg/mL streptomycin (Sigma).

### 3.4. MTT Assay

The 3-[4,5-dimethylthiazol-2-yl]-2,5-diphenyltetrazolium bromide (MTT) cytotoxicity assay is based on the ability of mitochondrial succinate dehydrogenase of viable cells to reduce the MTT tetrazolium salt HT22 cells were seeded in 96-well plates (8 × 10^3^ cells per well). After a 24-hour incubation of cells with 5 mM l-glutamate, MTT (final concentration = 0.5 mg/mL) was added and the cells were incubated at 37 °C for the next 4 h. Supernatants were then removed and dimethyl sulfoxide (DMSO) was added to dissolve MTT formazan crystals. The absorbance was recorded using a microplate reader (ELx800; BioTek Instruments, Inc., Seattle, WA, USA). The viability of cells treated with 5 mM l-glutamate was expressed as the percentage of the viability of control cells (untreated with 5 mM l-glutamate).

### 3.5. CO_2_ Measurement

HT22 cells were incubated in 5 mM sodium l-glutamate for 24 h. After treatment, the cells and supernatants were separated. Cells were then washed with a phosphate buffered saline (PBS) and suspended in a lysis buffer (0.15 M NaCl, 0.005 M EDTA, 1% Triton X-100, 0.01 M Tris-HCl). Next, sodium pyruvate was added (final concentration: 0.5, 1, 2, 3, 4, 5, 6, 7, 8, 9 and 10 mM) to the samples containing cell lysates and supernatants, respectively, prior to CO_2_ measurement. The CO_2_ level in each sample was assessed using a spectrophotometric stopped-flow technique. The cell-free complete culture medium was treated with 5 mM l-glutamate for 24 h and then collected. Next, a different concentration of sodium pyruvate (final concentration: 0.5, 1, 2, 3, 4, 5, 6, 7, 8, 9 and 10 mM) was added prior to CO_2_ measurement. The concentrations of CO_2_ and H_2_O_2_ were determined using the complex *cis*-[Cr(C_2_O_4_)(pm)(OH_2_)_2_]^+^ as the biosensor.

### 3.6. Instrumentation

The UV-visible spectroscopy studies were conducted using a Perkin-Elmer Lambda 18 Instrument with the scan accuracy of 1 nm and 1 nm slit width at a scanning rate of 120.00 nm min^−1^. Kinetic measurements were carried out using a stopped-flow technique and an Applied Photophysics SX-17MV spectrophotometer. The observable rate constants and concentrations of CO_2_ were computed based on the global analysis using a “Glint” program [25,26,27,28,29].

### 3.7. Statistical Analysis

Statistical analysis was performed using Statistica 9 software (StatSoft, Kraków, Poland). Data are expressed as mean considering SD (standard deviation). Statistical differences were evaluated using the Mann-Whitney U test. Differences were considered significant at *p* < 0.05, *p* < 0.01, *p* < 0.001.

## 4. Conclusions

In this paper an application of a new method for H_2_O_2_ detection in biological samples *i.e.*, hippocampal HT-22 cells, has been described. Moreover, the usefulness of this method, which is based on the interaction of CO_2_ with the coordination cation *cis-*[Cr(C_2_O_4_)(pm)(OH_2_)_2_]^+^ as a specific molecular biosensor, for the H_2_O_2_ detection in biological materials was discussed. The presented method turned out to be also handy tool to analyze the scavenging reaction of H_2_O_2_ by sodium pyruvate. Consequently, the efficiency of this scavenger was clearly demonstrated by the novel application of the H_2_O_2_ molecular biosensor*—cis-*[Cr(C_2_O_4_)(pm)(OH_2_)_2_]^+^ complex cation.
